# Development of a Microfluidic Chip Powered by EWOD for In Vitro Manipulation of Bovine Embryos

**DOI:** 10.3390/bios13040419

**Published:** 2023-03-25

**Authors:** Adriana Karcz, Ann Van Soom, Katrien Smits, Sandra Van Vlierberghe, Rik Verplancke, Osvaldo Bogado Pascottini, Etienne Van den Abbeel, Jan Vanfleteren

**Affiliations:** 1 Centre for Microsystems Technology (CMST), Imec and Ghent University, Technologiepark Zwijnaarde 126, 9052 Zwijnaarde, Belgium; 2 Reproductive Biology Unit (RBU), Department of Internal Medicine, Reproduction and Population Medicine, Faculty of Veterinary Medicine, Ghent University, Salisburylaan 133 D4, 9820 Merelbeke, Belgium; 3 Polymer Chemistry and Biomaterials Group, Centre of Macromolecular Chemistry, Ghent University, Campus Sterre, Building S4, Krijgslaan 281, 9000 Ghent, Belgium; 4 Department of Human Structure and Repair, Ghent University, Corneel Heymanslaan 10, 9000 Ghent, Belgium

**Keywords:** digital microfluidics, electrowetting on dielectric, cell manipulation, individual embryo culture, lab-on-a-chip

## Abstract

Digital microfluidics (DMF) holds great potential for the alleviation of laboratory procedures in assisted reproductive technologies (ARTs). The electrowetting on dielectric (EWOD) technology provides dynamic culture conditions in vitro that may better mimic the natural embryo microenvironment. Thus far, EWOD microdevices have been proposed for in vitro gamete and embryo handling in mice and for analyzing the human embryo secretome. This article presents the development of the first microfluidic chip utilizing EWOD technology designed for the manipulation of bovine embryos in vitro. The prototype sustains the cell cycles of embryos manipulated individually on the chips during in vitro culture (IVC). Challenges related to the chip fabrication as well as to its application during bovine embryo IVC in accordance with the adapted on-chip protocol are thoroughly discussed, and future directions for DMF in ARTs are indicated.

## 1. Introduction

Infertility, defined as the “failure to establish a clinical pregnancy after 12 months of unprotected intercourse” [1], concerns up to 12% of couples worldwide [2]. It is a condition in which the functionality of the reproductive system of an individual is impaired, which may have numerous causes. These include certain pathologies of the genital tract, past or ongoing general diseases, genetic predispositions as well as an unhealthy lifestyle [3,4,5,6]. Moreover, the origin of 15 to 30% of infertility cases remains unclear [7]. Furthermore, subfertility has numerous comorbidities and may lead to mental health disorders and psychological distress [8,9,10]. Partners suffering from reduced fecundity can turn to assisted reproductive technologies (ARTs) that were developed to facilitate conception and early embryo development, providing a potential solution to achieve pregnancy and the birth of a healthy offspring. Owing to the emergence and continued amelioration of ARTs, more than 10 million babies have been born worldwide since 1978 [11]. In humans, an in vitro fertilization (IVF) cycle involves hormonal stimulation of ovaries and subsequent retrieval of mature oocytes or in vitro maturation (IVM) of oocytes, in vitro fertilization (IVF) or intracytoplasmic sperm injection (ICSI), in vitro culture (IVC) of preimplantation embryos, and embryo transfer (ET) to the uterus. Despite the early unfolding of research that led to and shaped the current assisted reproductive procedures [12], the success rates of in vitro embryo production (IVP) are considered suboptimal and vary between species. In human IVP, approximately 50% of in vitro fertilized oocytes reach the blastocyst stage [13]. In animal IVP, better results can be achieved when the in vivo matured ovum is used as compared to IVM eggs. In mice and cattle, approximately 80–90% [14] and 55% [15] of zygotes can grow into blastocysts from matured oocytes collected from the uterine tubes, respectively. The results differ significantly when oocytes undergo IVM, decreasing the developmental rates to 38% [16] and 20–40% [17] in murine and bovine IVP, respectively.

In vivo, the mammalian oviduct provides conditions that are crucial for the creation as well as the development of preimplantation embryos [18]. Therefore, the processes leading to the fusion of gametes as well as the preimplantation embryo growth are mediated by the cascade of dynamic interactions between the gametes, an embryo, and the oviductal environment. Two types of cells build up the oviductal epithelium that define its functionality, i.e., the secretory and ciliated cells. Oviductal cilia are responsible for the pick-up of a cumulus oocyte complex (COC) from the ovarian follicle and its transfer to the ampullary region of the fallopian tube [19]. After coitus, ejaculated sperm cells travel through the uterine environment towards the isthmus of the oviduct, where the sperm reservoir is created. Increasing progesterone levels at the time of and after ovulation affect the motility and release of the capacitated spermatozoa from the reservoir. Hyperactivated sperms travel towards the ampulla where they meet a COC and fertilization occurs [20]. Secretory cells excrete the oviductal fluid composed of molecules that nourish the embryo at time of cleavage. Transport towards the uterus, where implantation takes place, is steered by the tubal flow and smooth muscle contractions. Additionally, the composition of the fluid is regulated in a dynamic manner by both the oviductal environment and the presence of a developing embryo to satisfy its energy requirements at the specific cleavage stages [21]. Such communication is entirely bypassed in in vitro settings, where early embryos are cultured singly or in groups in the “sea” of synthetic oviductal fluid, which is a watery-like culture medium, on a hard polystyrene Petri dish with no motion. Therefore, there is plenty of room for improvement of the in vitro assisted reproduction procedures that are currently in place. 

**Figure 1:**
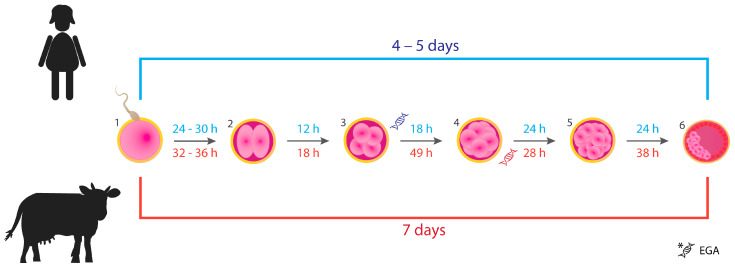
Timing of the early development of human (blue color) and bovine (red color) embryos from fertilization to the blastocyst stage. (1) Spermatozoon penetrates the zona pellucida of the oocyte: fertilization. (2) Zygote after the 1st cell division (2-cell). (3) Embryo after the 2nd cell cycle (4-cell). (4) Embryo after the 3rd cell cycle (8-cell). (5) Morula, embryo at 16- to 32-cell stage. (6) An early blastocyst: the inner cell mass (embryo proper) represented by the pink clustered cells will differentiate into structures that will give rise to a fetus. Trophoblasts surrounding the blastocyst cavity (depicted as red cells) will develop into the placenta. Stages at which embryonic genome activation (EGA) occurs in both species are shown. The figure was based on the information provided in articles [27,28,29,31,32,33].

The mouse is the most extensively used animal model in studies on mammalian reproduction. However, the existing genetic and morphological differences between mouse and human embryos entail the use of other mammalian species as models for study purposes as well as validation of novel methods in ARTs [22]. Cattle may provide a suitable model, as the early embryo development in vitro as well as the size of blastocysts more closely resemble the human species, with embryonic size being an important characteristic for microfluidic studies. Figure 1 presents similarities between the timing of subsequent cell division cycles (cleavage) from the point of fertilization until the blastocyst formation in human and bovine species. A bovine in vitro produced expanded blastocyst (at day 7 or 8 of culture) has a diameter of approximately 194 µm [23]. A human expanded blastocyst (at day 5) created using a donor oocyte, which underwent in vitro fertilization and culture has a diameter of approximately 194 µm [24]. For comparison, a murine expanded blastocyst (collected from a female mouse at day 3.5 post mating) has a diameter of 104 µm [25]. Embryonic genome activation (EGA) happens at different developmental stages and varies among species [26]. In humans, the EGA occurs at 4- to 8-cell stage [26,27,28,29]; however, earlier initiation of embryonic genome transcription was suggested and reported recently, indicating it is triggered after fertilization [29]. In cattle, the EGA has been reported to occur at 8- to 16-cell stage [28,30]; however, the state-of-the-art gene profiling of 1-cell zygotes may lead to similar observations as in humans. In vitro production of bovine embryos has played a significant role in the establishment of a genetic pool from superior individuals in beef and dairy cattle. Since 2016, a switch towards the use of IVP embryos instead of multiple ovulation embryo transfer among cattle breeders has been observed [17]. 

Bovine IVP plays an important role in the studies on reproduction, which led to the advancements in mammalian IVP. Moreover, it gives insight into the effects of environmental factors such as heat stress on early gestation. The availability of the inexpensive abattoir-derived material contributed to the increasing interest in bovine IVP [30,34,35]. In cattle, the conditions of IVC affect the timing of embryo development and the size of the resulting blastocysts [33]. The in vivo culture of IVP zygotes in the oviducts of other mammals or the homologous bovine resulted in better quality blastocysts, while the IVC of in vivo-derived zygotes yielded lower quality blastocysts [17,36]. Culturing bovine embryos in groups instead of as individual embryos [37] and addition of extracellular vesicles (EVs) collected from the female’s reproductive tract to the culture medium [38] were found to alleviate the detrimental effects related to the lack of the embryo-maternal crosstalk in in vitro settings. Therefore, it becomes clear that the inferior development of lab-grown mammalian embryos can be linked to the absence of the oviduct, as well as suboptimal culture conditions [30,36]. Despite various attempts to imitate the surroundings of a developing embryo in vivo, a culture system that would mimic such a sophisticated, dynamic microenvironment, is still lacking.

Microfluidics encompasses the study of the behavior and methods for manipulation of liquids in microstructured devices as well as their fabrication [39]. With its advancement, novel solutions for the on-chip manipulation of gametes and embryos were proposed and extensively reviewed in recent years [40,41,42,43]. Furthermore, microdevices that utilize electric phenomena, such as dielectrophoresis (DEP) and electrowetting on dielectric (EWOD), are being progressively recognized as promising tools in mammalian ARTs [44]. Electrowetting on dielectric technology, known as digital microfluidics (DMF), allows for the automation of laboratory workflows, and its field of application is continuously expanding [45,46]. The technology employs applied electric fields [47] to manipulate pico- to micro-liter (10^−12^–10^−6^ L) droplets in a rapid manner on an array of insulated electrodes on a chip [48,49,50]. Plentiful examples of EWOD-based microdevices created for chemistry and life sciences exist due to its applicability for handling of fluids of markedly lower volumes (when compared to pipetting) as well as the possibility of sensor integration within the chip [51,52,53,54,55,56,57]. The portability of EWOD devices is promising for the development of point of care diagnostics [53]. These microdevices can be equipped with components allowing for the control of pH or temperature, as well as detection of electrochemical, optical, and electrical signals [58]. Moreover, incorporation of hydrogels can be used for vertical transport of fluids [59], while on-chip gelation provides a platform for three-dimensional adherent cell culture [60]. The combination of magnetic beads and EWOD has proved useful for the analysis of spent embryo culture medium, and isolation of DNA and biomarkers has been reported [61,62,63]. The latter is exceptionally appealing for assisted reproduction because two molecules were detected simultaneously from medium collected during single human embryo culture, which is unachievable with standard techniques such as enzyme-linked immunosorbent assay due to sample volume requirement. Interestingly, this analysis uncovered that single human embryos collected from the same patient excreted various levels of these molecules, providing a tool for the assessment of an individual embryo’s developmental competency. Droplet operations such as merging and splitting could serve as a tool to provide the dynamic culture conditions, medium supply, and/or refreshment as well as delivery of specific molecules such as growth factors and microRNAs required at a specific time of preimplantation embryo development, thereby truly mimicking the embryo-maternal dialogue in vitro. Ideally, such microdevices will comprise sensors allowing for the evaluation and maintenance of the on-chip microenvironment of an early embryo produced in the laboratory. However, the application of digital microfluidics in ARTs is in its infancy, and thus far only murine embryos were successfully manipulated and cultured on a chip [64]. The novelty of advanced technologies necessitates validation in various animal studies, which could likewise benefit their commercial application in the near future. Differences in early embryonic development between the species exist, which are significant for the construction of microfluidic devices for embryo culture in vitro. Here, we demonstrate the development of the first digital microfluidic device suitable for the in vitro manipulation of bovine embryos. The process of fabrication and the first application in bovine IVP are presented. Encountered challenges and future directions for chip development are identified and discussed.

## 2. Materials and Methods

### 2.1. Materials and Chemicals

#### 2.1.1. Microdevice Fabrication

One and two inch square Indium Tin Oxide (ITO) coated borosilicate glass substrates (CEC020B) and 2 inch square borosilicate glass substrates were purchased from Präzisions Glas & Optik GmbH (Iserlohn, Germany). Microposit S1818 positive photoresist and Microposit developer concentrate were purchased from micro resist technology GmbH (Berlin, Germany). Silane (SiH_4_), nitrogen (N_2_) and nitrous oxide (N_2_O) were purchased from Nippon Gases (Schoten, Belgium). Ammonia (NH_3_) was purchased from Air Products (Allentown, PA, USA). The above-mentioned gases, SiH_4_, N_2_, NH_3_, and N_2_O, were used for the Plasma Enhanced Chemical Vapor Depositions (PECVD) of silicon (IV) nitride (III) (SiN_x_), referred to as “silicon nitride”. Water vapor (99.999% pure) and trimethylaluminum (TMA, 99.999% pure) were purchased from Strem Chemicals (Newburyport, MA, USA) and served as precursors for the Atomic Layer Deposition (ALD) of aluminum (III) oxide (II) (AlO_x_), referred to as “aluminum oxide”. Nitrogen (N_2_), used as purge and carrier gas during ALD, was purchased from Nippon Gases (Schoten, Belgium). Fluoropel PFC1601V-FS, further referred to as ”Fluoropel”, was purchased from Cytonix (Bestville, MD, USA). Polyimide Film Tape (5413) and High Performance Double Coated Tape (9088-200) were purchased from 3M (Saint Paul, MN, USA). Conductive carbon double sided adhesive was purchased from SPI supplies (West Chester, PA, USA). All other chemicals used during processing of the chips were purchased from VWR (Leuven, Belgium) unless stated otherwise in the text.

#### 2.1.2. In Vitro Production of Bovine Embryos

Tissue Culture Medium TCM-199, gentamycin, and kanamycin were purchased from Life Technologies Europe (Ghent, Belgium). Phosphate Buffered Saline (PBS), 4-well and 35 mm culture dishes were purchased from Gibco^TM^ Thermo Fisher Scientific (Waltham, MA, USA). Percoll was purchased from GE Healthcare Biosciences (Uppsala, Sweden) and paraffin oil for tissue culture was obtained from SAGE In Vitro Fertilization (Cooper Surgical Company, Trumbull, CT, USA). Tween80 (Uniqema Americas LCC) was purchased from Cospheric LCC (Goleta, CA, USA). All other chemicals used for the preparation of the culture media were purchased from Sigma-Aldrich (Diegem, Belgium) unless stated otherwise. All media were filtered using 0.2 µm filters from Pall Corporation (Ann Arbor, MI, USA). 

### 2.2. Methods

#### 2.2.1. Fabrication of EWOD Chips 

**Figure 2:**
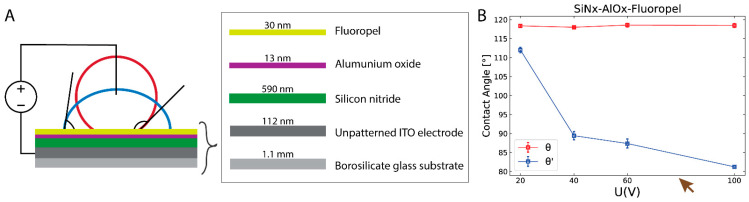
Evaluation of the electrowetting on dielectric (EWOD) phenomenon on a microfluidic platform comprising a multilayer dielectric hydrophobized with Fluoropel upon the application of voltage. (**A**) A schematic representation of the change of the static contact angle (SCA) of the unpotentiated aqueous droplet (red line) in response to the applied electric field (blue line). The bottom substrate with deposited insulating and hydrophobic coatings is shown. (**B**) Initial (*θ*, no voltage applied) and potentiated SCAs (*θ*′, after the application of DC voltages ranging from 20 V to 100 V) of 5 µL PBS droplet are presented. The brown arrow indicates a measurement point at which electrolysis occurred upon the application of 80 V on one of the samples (1/3). Reproduced in part with permission from the Chemical and Biological Microsystems Society (CBMS). Copyright 2020 CBMS [65].

**Figure 3:**
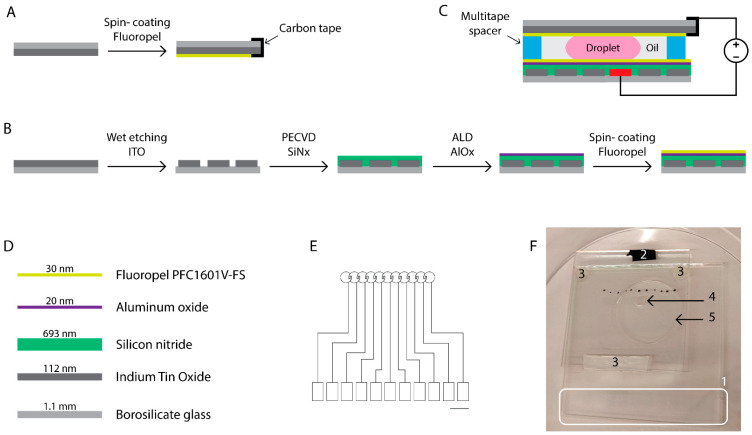
Development of the chip for the in vitro manipulation of bovine embryos. Schematic representation of the fabrication of the top (**A**) and bottom (**B**) plates. The top electrode (**A**) that was connected to the ground with conductive carbon tape is flipped and attached to the bottom plate, which is processed as shown in (**B**), using multitape spacers as depicted in (**C**). The droplet of culture medium is sandwiched between the plates and surrounded by oil. The bottom plate comprising the driving electrodes consisted of the materials presented in (**D**). The design of the electrodes that are projected and patterned onto glass substrates coated with conductive Indium Tin Oxide (ITO) is shown in (**E**). Black scale bar = 5 mm. The rectangular pads depict the ITO contact pads, which were protected from the deposition of the insulating and hydrophobic coatings during processing. The voltages were applied by placing the source electrode in contact with the rectangular contact pad, providing the electric field to the desired circular electrode on top of which the culture medium droplet was residing. (**F**) Picture of the actual microdevice with its components: (1) ITO contact pads, (2) conductive carbon tape used to connect to the ITO ground electrode on the top substrate, (3) multitape spacers distancing the plates of the chip, (4) a 5 µL droplet of culture medium, and (5) 100 µL of surrounding oil. Black dots mark the location of the driving electrodes for facilitation of droplet actuation.

In this section, the process parameters used for the fabrication of the EWOD-based microsystems are provided. The process flows employed in the manufacturing of different versions of the platforms will follow in the text. Additionally, Figure 2 and Figure 3 depict the detailed information on the coatings included in the chips with unpatterned ITO electrodes and patterned ITO electrodes intended for bovine embryo handling, respectively. Ellipsometry (FS-1EXs, FilmSense, Lincoln, NE, USA) was used to measure the thicknesses of aluminum oxide and Fluoropel. Spectral reflectance (F40-UV, Filmetrics, San Diego, CA, USA) was used to measure the thickness of silicon nitride. These measurements were performed on Si reference samples, which were included during the depositions. 

Development of the “open” chip with an unpatterned ITO electrode (Figure 2): 1″ and 2″ square ITO-coated borosilicate glass substrates were cleaned using a strongly diluted RBS T 105 detergent in deionized water (diH_2_O), isopropyl alcohol (IPA) and diH_2_O with ultrasonic agitation (Bransonic 5210, Branson Ultrasonics Corporation, Brookfield, CT, USA). The substrates were dehydrated on a hotplate (30 min at 150 °C) and exposed to oxygen plasma (5 min, 0.8 mbar, 190 W) before the deposition of the first dielectric layer. A small piece of the ITO electrode was covered with polyimide film tape during processing and removed before use. Advanced Vacuum Vision 310 PECVD (Plasma-Therm, Saint Petersburg, FL, USA) was used for the deposition of silicon nitride from a mixture of 100% SiH_4_ (40 sccm), N_2_ (1960 sccm), and NH_3_ (35 sccm) using high (13.56 MHz, 30 W) and low (100 kHz, 50 W) plasma frequencies. The pressure was set to 650 mTorr, and the process was performed at 250 °C. Two additional steps of samples’ exposure to oxygen plasma (24 s, 0.8 mbar, 190 W) were performed before the deposition of the subsequent layers of aluminum oxide and Fluoropel. Aluminum oxide was deposited with Atomic Layer Deposition in Ultratech Savannah S2000 G2 reactor (Cambridge NanoTech, Cambridge, MA, USA) using TMA (0.03 s followed by 30 s N_2_ purge) and H_2_O (0.03 s followed by 30 s N_2_ purge) pulsed intermittently at 150 °C and 53 kPa. Fluoropel (0.2 µm filtered) was spincoated for 30 s at 3000 rpm at an acceleration rate of 1500 rpm/s (Polos SPIN200i) and cured at 180 °C for 30 min on a hotplate. The change of the initial static contact angle (*θ*, measured before the application of the potential) of a 5 µL droplet of PBS upon the application of DC voltages ranging from 20 to 100 V in 20 V intervals in air environment was evaluated (*θ*′, measured after the application of voltage). The droplets were grounded using a copper wire inserted in the droplet. The current was monitored upon voltage application and when exceeding 1 nA, the current leakage was considered problematic. A Keithley 2400 Source Measure Unit (SMU, Tektronix UK Ltd., Bracknell, UK) was used to simultaneously provide the electric potentials and monitor the current. Measurements were conducted in 3 replicates, i.e., a 5 µL droplet was deposited once onto 3 separate samples, the *θ* as well as *θ*′ were measured for each droplet using the EasyDrop Drop Shape Analysis system (Krüss GmbH, Hamburg, Germany). The plot (Figure 2B) was made with Python (version 3.7.3) using anaconda (release 4.6.11). The matplotlib module with version 3.0.3 was used, and data was manipulated using NumPy module with version 1.16.2. 

Fabrication of the chip for bovine embryo manipulation is depicted in Figure 3: 2″ square ITO-coated borosilicate glass substrates were diced into 4 cm × 4 cm squares and used for the fabrication of the top plates that served as ground electrodes (Figure 3A). The samples were cleaned and coated with Fluoropel as described above. During spincoating of Fluoropel, a small piece of ITO was covered with scotch tape, which was removed before curing. Upon chip assembly, a piece of conductive carbon tape was attached to the uncoated piece of the ITO ground electrode enabling contact with the ground electrode, as indicated in Figure 3C. Undiced 2″ square ITO-coated borosilicate glass substrates were used as bottom plates (Figure 3B) and were cleaned as described above. Electrodes were patterned using a sequence of photolithography and wet etching. Briefly, positive photoresist S1818 was spincoated on the ITO-coated glass samples that had been exposed to O_2_ plasma (24 s, 0.8 mbar, 190 W). The samples were soft baked (2 min, 90 °C) and exposed to UV light through a glass mask containing the pattern of the electrodes. Next, the samples were submerged in the photoresist developer solution. The photoresist on the illuminated zones of the photoresist was dissolved in the solution exposing the underlying ITO, while the photoresist on the non-illuminated zones remained on the substrates protecting the underlying ITO. Then, the substrates were rinsed with diH_2_O and dried using a nitrogen gun followed by the hard baking step (30 min, 120 °C). The ITO was etched for 4 min 30 s at 45 °C in a mixture of H_2_O:HCl (32%):HNO_3_ (65%). The design comprised a path of 11 circular electrodes with 1500 µm radii. Three additional steps of samples’ exposure to oxygen plasma (24 s, 0.8 mbar, 190 W) were performed before the deposition of the subsequent SiNx, AlOx, and Fluoropel coatings. Deposition of SiNx as the first dielectric layer was achieved using PECVD as described above. The diverse contact pads were covered with polyimide film tape, which was removed before ALD deposition of AlOx as the second dielectric at 120 °C, in accordance with the procedure described above. The AlOx deposited on the contact pads was etched using a solution of sodium hydroxide (NaOH) in diH_2_O adjusted to a pH value of 12. Fluoropel was then spincoated on top of the AlOx layer as described above. Multilayered High Performance Double Coated Tape was used to create the 600 µm thick spacers distancing the top and bottom plates. The assembly process is presented schematically in Figure 3A–C.

#### 2.2.2. In Vitro Production of Bovine Embryos

**Figure 4:**
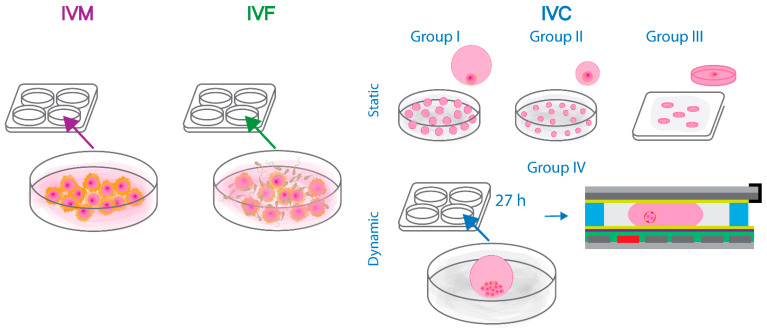
Schematic representation of the design of the static and dynamic bovine embryo culture experiments. Briefly, abattoir-derived oocytes were in vitro matured and fertilized in accordance with the standard protocols. Next, the presumed zygotes were divided into groups: I–III of statically cultured embryos and group IV in which bovine embryos were manipulated and assessed every 24 h on the developed digital microfluidic chips. The methods of in vitro maturation, fertilization, and culture of bovine embryos in groups I–IV are described in detail in the dedicated Materials and Methods section.

In vitro production of bovine embryos was performed in accordance with the procedure described in Ref. [66]. Briefly, abattoir-derived bovine ovaries were collected and processed within two hours. The gonads were wiped superficially with ethanol (96%) and triple rinsed in kanamycin enriched PBS (50 mg/mL) at 37 °C. A 18-gauge needle and 10 mL syringe were used to aspirate the cumulus oocyte complexes from 4 to 8 mm diameter follicles. Selected COCs characterized by compact cumulus and uniform cytoplasm were washed using HEPES-TALP medium and cultured in groups of 60 per 500 µL of modified bicarbonate-buffered TCM-199 medium supplemented with gentamycin (50 mg/mL) and epidermal growth factor (EGF, 20 ng/mL). After 22 h of in vitro maturation in 5% CO_2_ at 38.5 °C, the COCs were fertilized using proven bull spermatozoa. Frozen-thawed bull semen was segregated using a 45:90% Percoll gradient. The pellet containing viable spermatozoa was then washed in IVF-Tyrode’s-albumin-lactate-pyruvate (IVF-TALP) medium with bicarbonate-buffered Tyrode solution. The final concentration of spermatozoa was adjusted to 1 × 10^6^ cells per 1 mL of IVF-TALP medium supplemented with Bovine Serum Albumin (BSA, 6 mg/mL) and heparin (25 µg/mL). In vitro matured bovine oocytes were washed in 500 µL HEPES-TALP medium and co-incubated with spermatozoa for 21 h in 500 µL of IVF-TALP. After IVF, the presumed zygotes were vortexed to remove the surrounding cumulus cells and further handled depending on the experimental group (I–IV). A schematic representation of the experimental design is provided in Figure 4.

Single bovine embryos were cultured statically in 20 µL (group I, control, *n* = 65) or 5 µL (group II, *n* = 48) droplets of synthetic oviductal fluid supplemented with essential and non-essential amino acids (SOFaa), insulin (5 µg/mL), transferrin (5 µg/mL), selenium (5 ng/mL), and BSA (4 mg/mL) under 7.5 mL oil overlay in 5% CO_2_, 5% O_2_, and 90% N_2_ at 38.5 °C. Group III consisted of embryos cultured individually in 5 µL droplets of culture medium enriched with Tween 80 surfactant (0.01% *v*/*v*) surrounded by 400 µL of oil. The droplets were squeezed between two borosilicate glass plates hydrophobized with Fluoropel separated using 600 µm thick spacers (*n* = 17). Bovine embryos assigned to group IV were cultured for 27 h in groups of 22–28 in 50 µL droplets of SOFaa-ITS-BSA medium under 900 µL oil overlay. At 48 h post insemination (hpi) 2- to 4-cell stage zygotes from group IV were selected and washed in the SOFaa-ITS-BSA medium supplemented with Tween 80 (0.01% *v*/*v*). The procedure of the loading of a 5 µL droplet of culture medium enriched with Tween 80, which contained a single bovine zygote between the bottom and top plates of the chip, is shown in Supplementary Video S1. A total of 15 embryos were suspended individually in 5 µL droplets of SOFaa-ITS-BSA-Tween 80 medium, which were pipetted onto EWOD chips surrounded by 100 µL of oil. The proof-of-concept experiment was divided into two parts. In the first examination, 8 embryos were moved on the chip under a microscope every 24 h by the manual charging of the next pair of electrodes (Supplementary Video S2). In the second part, 7 embryos were moved in 5 µL droplets and supplied with embryo culture medium during IVC. Briefly, on five of the chips (5/7), a 2 µL droplet of pre-equilibrated medium was pipetted onto the chips (similar to embryo-droplets loading) at 72 hpi. After 24 h, droplets containing embryos were moved towards the on-chip equilibrated 2 µL droplets and merged by contact (the procedure is shown in Supplementary Video S3). On one of the chips, the droplets merged immediately as they were placed in close proximity. On two chips (2/7), 2 µL droplets were deposited in the vicinity of the oil/air interface, which led to their evaporation overnight. In these cases, at 96 hpi, a 5 µL pre-equilibrated droplet of SOFaa-ITS-BSA-Tween 80 medium was loaded on the chips, moved towards the embryo-containing droplet, and merged instantly creating a 10 µL droplet. Droplet manipulation was achieved by the application of 70–90 V. In the second part of the dynamic droplet manipulation experiment, potentials were occasionally increased to 100 V to move droplets of increasing volume. Videos available in the Supplementary material as well as pictures of embryos cultured in groups I–IV were obtained using ToupLite software (ToupTek Photonics Co., Hangzhou, China). The development of embryos cultured statically in groups I–III was assessed by morphological grading under stereo microscope at 48 and 192 hpi. Cleavage (at 48 hpi) and blastocyst rates (at 192 hpi), calculated as percentage of cleaved embryos, were analyzed with logistic regression models using R-core (version 4.1.2, R Core Team, Vienna, Austria) with the replicates set as random effect (group I was included in 4, and groups II and III in 3 replicates). Tukey’s post hoc test was used to evaluate the differences between the culture groups. The level of significance was set at *p* ≤ 0.05, and results are presented as least square means with their respective standard errors.

## 3. Results

### 3.1. Development of EWOD Microdevices with Multilayered Insulators

Electrowetting on dielectric technology is based on the electrowetting phenomenon and refers to the change of the contact angle between the conductive liquid and the electrode under the influence of an applied electric field [47]. In EWOD, an aqueous droplet is deposited on top of a dielectric that insulates the electrode. Three interfaces between the phases can be distinguished, namely the solid-vapor, solid-liquid, and liquid-vapor. Electrowetting on dielectric actuation is described by the Young-Lippmann Equation (1), which relates the interfacial tensions between these phases with the change of the surface energy caused by the application of an electric field. When voltage is applied, a charge imbalance is created in the dielectric, which alters the interfacial energy. As a consequence, the apparent contact angle (*θ*) between the droplet and the solid surface is modulated (*θ*′). The Young-Lippmann equation is expressed by:

(1) $$cos\theta^{\prime }=cos\theta +\frac{1}{2}\frac{cV^{2}}{\gamma_{lv}},$$

where *θ*′ and *θ* are the potentiated and initial contact angles, respectively, *c* is the capacitance of the dielectric $\left[\frac{F}{m^{2}}\right]$, *V* is the applied potential, and *γ_lv_* is the liquid-vapor interfacial tension $\left[\frac{10^{-3}N}{m}\right]$. 

The results of the application of DC voltages on the EWOD response in the system comprising a multilayered dielectric, which consists of the stack as shown in Figure 2A (SCA = 118° ± 0.5°), are shown in Figure 2B. Information about the materials used for the system development are listed in Table 1. Leakage currents at a value 1 nA or larger are considered to be relevant for the operation of the device intended for bovine embryo manipulation. 

### 3.2. In Vitro Development of Bovine Embryos: Static Culture and Dynamic Manipulation on the Developed Chips

**Figure 5:**
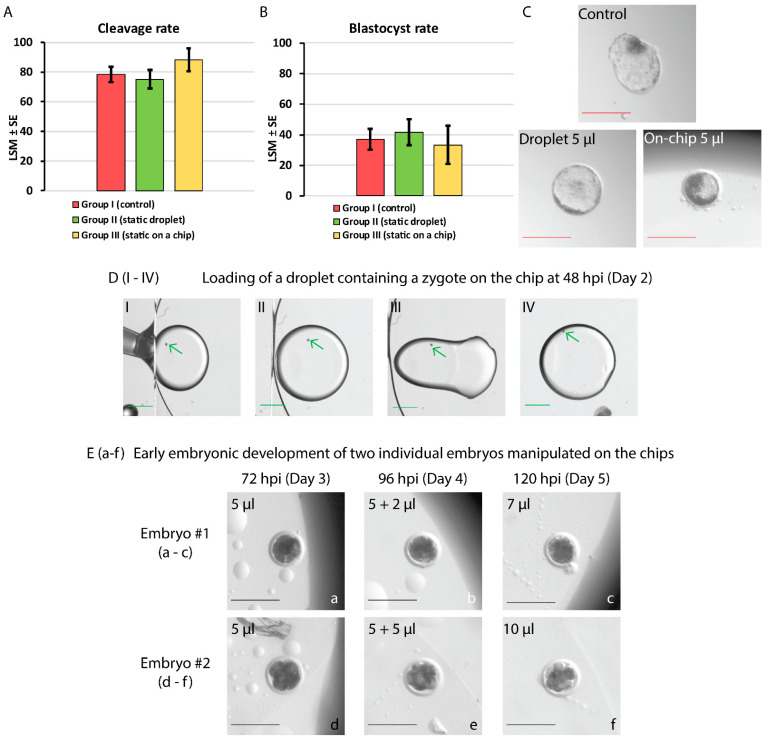
Static in vitro culture (**A**–**C**) and on-chip manipulation of bovine embryos (**D**,**E**). (**A**,**B**) show the cleavage and blastocyst rates of embryos cultured in groups I–III assessed at 48 and 192 hpi, respectively. No statistically significant differences in cleavage and blastocyst rates among the groups were found (*p* > 0.05). (**C**) depicts the in vitro embryo development at 192 hpi (day 8): a hatching blastocyst in the control (group I), an expanded blastocyst in a 5 µL droplet on the Petri dish (group II), and an early blastocyst in a 5 µL droplet sandwiched on the chip (group III). Red scale bar = 200 µm. (**D**) Frames showing embryo loading on the chip at 48 hpi (DI–DIV). The edge of the top plate (ground electrode) as well as oil/air interface are visible in DI–DIII. Briefly, an embryo-containing droplet of culture medium enriched with Tween 80 (0.01%) is pipetted between the bottom and top plates of the chip (DI–DII). Next, the droplet is moved away from the edge and air/oil interface by the application of 70 V to the bottom driving electrode while the top electrode is grounded (DIII–DIV). Green arrows indicate the location of the zygote. Green scale bar = 1000 µm. (**E**) depicts two embryos (a–c and d–f) manipulated on the chip and supplied with a 2 or 5 µL droplet of medium merged with the embryo-containing droplet at 96 hpi. Cleavage of two 4-cell embryos is presented until 120 hpi (day 5 of culture). Black scale bar = 200 µm.

Cleavage and blastocyst rates achieved after static IVC of bovine embryos assigned to groups I–III, assessed at 48 and 192 hpi, respectively, are shown in Figure 5A,B.

No statistically significant differences in the embryonic development (proportions of cleaved embryos or blastocysts) among these groups of embryos cultured individually were found (*p* > 0.05). Although group III is presented next to groups I and II, due to the diminished number of embryos assigned within, this result shall be considered an indication at present. Figure 5C shows pictures of individual blastocysts at 192 hpi from each group (I–III).

In the proof-of-concept experiment, two modes of embryo manipulation on the developed EWOD devices were tested. In total, 15 bovine embryos at 2- to 4-cell stage were selected at 48 hpi from a total of 77 embryos (cleavage rate of 86% ± 7.4%). Table 2 shows the development of bovine embryos at the time of assessment every 24 h by morphological grading. Corresponding duration of subsequent cell cycles in bovine species, as shown in Figure 1, are included. The number (and percentage %) of embryos at relevant times of development are listed. Pictures of two embryos taken during EWOD manipulation until 120 hpi (between days 3 and 5) are presented in Figure 5E (a–c and d–f). In total, 2 out of 15 dynamically manipulated embryos grew until the morula stage.

## 4. Discussion

### 4.1. Development of EWOD Chips Using Multilayered Dielectric Approach

For the manufacturing of an EWOD microsystem, the choice of a suitable dielectric layer becomes crucial as its properties determine the range of applicable voltages allowing for the successful droplet movement. In general, low voltage electrowetting is preferred, especially when handling biological samples containing living cells. Voltage requirements to achieve surface wetting can be decreased by reducing the thickness of the insulator. This approach, however, may lead to the failure of the EWOD microsystem due to electrical breakdown of the insulator upon the application of an electric field. Additionally, if defects such as pores are present in the dielectric, the conducting liquid may propagate through the layer, damaging the underlying electrode. Schultz et al. suggested the introduction of a multilayered dielectric consisting of different materials to reduce the possibility for the above-mentioned effects to occur [70]. Here, we proposed the addition of a nanofilm of high-quality ALD aluminum oxide (13 nm) on top of PECVD silicon nitride (590 nm). Ideally, thanks to the utilized electrical insulators, no DC current is expected to flow between the electrodes when applying a DC voltage. In reality, however, small leakage currents do flow between these electrodes. Monitoring of the current can be considered as a harbinger of the dielectric failure, which may lead to the degradation of the device. When such breakdown occurs, a sharp increase in the leakage current can be observed [71]. Such gradual as well as spontaneous discharge of energy stored within the insulator could be harmful to the exposed cells. Although little is known about the effects of the applied potential on the development of bovine embryos, mouse and rat early embryos were found to be sensitive to the external electric fields in vitro. The results differed between the two species in terms of stages of embryonic development and the strength of the applied electric fields [72]. Additionally, changes in the membrane potentials in mouse oocytes and embryos throughout the gamete and early embryonic development have been reported [73]. Bioelectric signals are involved in the processes such as migration of cells, wound healing, and embryogenesis among various species [74,75,76]. Endogenous electric fields are involved in the development of amphibian embryos; however, disruptions in these internally generated electric fields lead to morphological abnormalities. Xenopus embryos exposed to currents between 100 nA and 500 nA showed various degrees of visible deformities [77]. Therefore, in this study, a current value of 1 nA was considered relevant for the manipulation of droplets containing living multicellular organisms, such as mammalian embryos, in the attempt to exclude its possible harmful effect on their development in vitro. The results of the application of DC potentials (20–100 V) on the proposed open microsystem are presented in Figure 2. In general, by using the multilayered dielectric approach, two beneficial effects were achieved: (1) significantly enhanced range of operational voltages and (2) avoidance of current leakage. In our study, no plots were obtained when 590 nm silicon nitride was used as a dielectric alone (hydrophobized with 30 nm Fluoropel) as current leakage, and electrolysis occurred already at voltages as low as 30 V. 

Silicon nitride and aluminum oxide are characterized by moderately high dielectric constants of approximately seven and nine, respectively. Silicon nitride deposited by PECVD can be characterized by conformal coating, high breakdown field, and low current leakage. Atomic layer deposition allows for the deposition of highly conformal ultra-thin layers (below 10 nm thick). Due to the properties of ALD aluminum oxide, such as the amorphous structure and large band gap, low current leakage and good adhesion can be achieved. Additionally, both materials can be deposited at relatively low temperatures [67,78,79]. In a prior study of the stacked dielectrics, with the introduction of an ALD aluminum oxide thin film atop four materials common in microelectromechanical systems fabrication, these beneficial effects were observed and confirmed [65]. Upon chip fabrication (intended for the use with bovine embryos), both dielectrics were deposited at longer times (as compared to the open devices), which resulted in thicker coatings (Table 1) to ensure stable operation in the range of expected DC potentials required for complex fluid manipulation. At the resulting thicknesses within the EWOD microsystem (<1 µm), the chips are fully transparent, which allowed for evaluation of the on-chip embryo growth using an optical microscope (Figure 3). Droplets of SOF-ITS-BSA Tween 80 enriched medium of 5 µL, each containing a single embryo, were moved from one electrode pair to the next one at 70–90 V. In this design, the volume of the surrounding oil was reduced to 100 µL.

### 4.2. Culture Medium Modification with Tween 80 

Bovine embryo culture medium is a complex fluid composed of essential nutrients that sustain the subsequent cellular divisions during early embryogenesis in vitro. Apart from glucose, pyruvate and lactate, organic acids, amino acids, electrolytes and metal ions, SOF is supplemented with BSA and insulin, transferrin and selenium, which particularly favors individual bovine embryo culture [80]. Bovine serum albumin acts as an anti-oxidant and a protective and carrier agent due to its binding ability to various ligands, including molecules that mediate cell growth [81]. However, the presence of proteins within a droplet poses a challenge for digital microfluidics due to the undesired adsorption of molecules onto surfaces [82]. Fouling with proteins affects the surface tension, which in turn hinders the EWOD actuation, even after short incubation times. The addition of surfactants is a common method used to overcome this problem [83]. Tween 80 belongs to a group of compounds (Tween 20, 40, 60, 80), which contain an ethylene glycol hydrophilic head and a hydrophobic tail that differs between the types. Coating the surfaces with Tween 80 surfactant renders them resistant to protein adsorption due the high affinity to low energy surfaces. Additionally, Tween 80 reacts with BSA forming association complexes in aqueous solutions and supports the protein stability [84,85,86]. Nonetheless, any attempt of culture medium modification raises concerns about cytotoxicity. For this reason, an additional small-scale test was conducted, which indicated that SOFaa-ITS-BSA with 0.01% Tween 80 did not have a harmful effect on the development of bovine embryos (Supplementary Material: Tables S1 and S2, and Figure S1).

### 4.3. In Vitro Culture and On-Chip Manipulation of Bovine Embryos

Microfluidics allows for a significant reduction of the amounts of handled fluids. However, decreasing the volume is challenging in standard embryo IVC. Inaccurate pipetting of medium during culture dish preparation may lead to instabilities such as modified droplet shape and/or increased osmolarity upon rapid evaporation, which may be harmful to developing embryos [87]. Therefore, in the static embryo culture experiment, three groups were designed to investigate the effects of the changed culture conditions, i.e., decreased volume (5 µL) on a Petri dish and “squeezed” droplets (5 µL sandwiched between two glass plates). Although no differences were found in cleavage and blastocyst rates among the three groups, embryos cultured “on-chip” (group III) showed signs of growth retardation, reaching only the early blastocyst stage at day 8 (192 hpi) compared to the hatching and expanded bovine blastocysts in groups I and II (Figure 5C). This could be explained by the disrupted diffusion of molecules and products of embryo metabolism in medium droplets of modified shape. Ammonium, which gradually accumulates within embryo surroundings during IVC, was found to alter the gene expression of human and mouse blastocysts leading to delayed embryo growth [88]. Exposure of bovine ova to ammonium resulted in an increased number of degenerated embryos and decreased rates of blastocyst formation [89]. It should be noted, however, that the number of embryos allocated to group III was markedly lower than in groups I and II. Thus, it is recommended that these results are followed by additional experiments and should be treated as an indication solely under the current circumstances.

The experimental plan for the on-chip embryo treatment assumed that it would be possible to (1) mix the components of the growth medium and potentially harmful products of cell metabolism within the droplet and to (2) supply fresh medium during culture. Nonetheless, due to the limitations of the developed EWOD devices, i.e., the necessity for manual application of electric signals, the chips were removed from the incubator to drive the droplets. This posed additional challenges for the early embryo growth due to frequent exposure to non-biomimetic conditions such as atmospheric oxygen, light and variations in temperature [90]. Reactive oxygen species (ROS) are crucial in mediating cellular functions and are involved in pathway signaling during embryonic development in bovine species [91]. Oxidative imbalance, however, has detrimental effects on these mechanisms. The excess of ROS during mammalian IVC may originate from endogenous and exogenous processes such as oocyte and embryo metabolism, and interactions with the embryo’s surrounding, i.e., the culture medium and its handling, respectively [92]. Moreover, lowering the oxygen concentration (1%) results in significantly higher blastocyst rates in bovine embryos, especially favorable when cultured individually [93]. Exposure to light has been reported to affect the early embryonic development in vitro and protein expression among mammalian species [90,94]. Additionally, excessive display of the medium or oil to light may lead to the photooxidation of their compounds [95]. Although little examination of the effects of daily exposure of bovine embryos during IVC to temperature variations on the developmental rates exist, undisrupted culture conditions are preferred, which are compatible with the time-lapse system [96]. Recently, the effects of the incubation manner on the development of IVP human embryos were evaluated. Eggs were cultured in the benchtop incubator or using the time-lapse system. The former required daily removal of culture dishes for scoring and/or media change (days 1–3), similarly to our experimental procedure, whereas in the latter only for media change (day 3). A significantly higher rate of blastocyst formation was observed among IVF embryos cultured using the time-lapse system when compared to the benchtop incubator [97]. Based on the above-mentioned findings, to our mind, these stress factors might have had synergistic, adverse effect on the on-chip cultured bovine embryos, which led to developmental delay, arrest, or degeneration. 

During experiments involving manipulation of embryos in droplets on the chips, an important observation was made: the deterioration of the adhesive properties of the spacers upon prolonged exposure to humidity was impairing the droplet driving (day 5 vs. day 8). This challenge can be solved by the introduction of a covalently bonded spacer, for instance, polydimethylsiloxane (PDMS) and glass using O_2_ plasma, a common method in microfabrication [98]. Thus, it is the authors’ recommendation that for incubation in a humidified atmosphere lasting as long as a few days, the material distancing the driving and ground electrodes in a “sandwiched” EWOD chip configuration is replaced. This, together with a setup allowing for droplet manipulation from outside of the incubator, can be easily adapted to serve as the first digital microfluidic microdevice for the in vitro culture of bovine embryos. In addition, the developed microdevices can be evaluated for in vitro handling of mammalian gametes, namely maturation and fertilization, in the current form. 

## 5. Conclusions

Microfluidics allows for a better approximation and monitoring of the conditions of the cellular microenvironment in vivo. Although the potential of the use of (D)MF in ARTs seems eminently promising, due to the novelty of this research field, more studies need to be performed to evaluate the mode of application as well as its effects on the development of mammalian embryos. In this article, the results of the static bovine embryo culture in vitro along with the effects of the first application of the developed EWOD chip are presented. Future perspectives for the prototype optimization are indicated. Although the desired result, i.e., the on-chip embryo culture until the blastocyst stage, was not yet achieved, two modes of successful embryo-containing droplet actuation are demonstrated: droplet transport and medium supply during IVC. Additionally, various effects of the adapted protocol are evaluated during static individual embryo culture. These results showed that decreasing the volume of the culture medium did not affect blastocyst rates in droplet embryo culture, indicating the direction towards the development of more biomimetic IVP microdevices for ARTs. Furthermore, early bovine embryos that were manipulated by EWOD seem to have survived the applied electric fields. Digital microfluidics has the potential to make a true lab-on-a-chip possible, and its application in ARTs will drive the long-awaited advancements of methods for the in vitro handling and analysis of gametes and embryos.

## Figures and Tables

**Table 1:** Thicknesses and dielectric constants for the different dielectric and hydrophobic layers of materials used for EWOD devices.

Coating	Material	Thickness		Dielectric Constant *ε_r_* *
		Open Platform (Figure 2)	Chip for Embryo Manipulation (Figure 3)	
Dielectric 1	Silicon nitride	590 nm	693 nm	6–8 [67]
Dielectric 2	Aluminum oxide	13 nm	20 nm	9 [68]
Hydrophobic **	Fluoropel PFC1601V-FS	30 nm	30 nm	1.9 [69]

* ε varies depending on the deposition method. Given values ([67,68,69]) can be used as reference. ** Hydrophobic layers are usually very thin and are characterized by low *ε_r_*. Thus, they do not contribute markedly to the voltage calculations in the Young-Lippmann equation.

**Table 2:** Numbers and percentages (in brackets) of bovine embryos manipulated individually on the chips with the timing of the corresponding cell division cycles until blastocyst formation.

Morphological Assessment		48 hpi	72 hpi	96 hpi	120 hpi	144 hpi
Corresponding Cell Cycles (Timing in Hpi)		1st (32–36) 1-Cell to 2-Cell	2nd (36–54) 2-Cell to 4-Cell	3rd (54–103) 4-Cell to 8-Cell	4th (103–131) 8-Cell to Morula	Blastocyst Formation (131–192)
**Developmental stage**	2-cell	7 (47%)				
	4-cell	8 (53%)	12 (80%)	10 (67%)		
	8-cell		2 (13%)	4 (27%)	2 (13%)	
	Morula				2 (13%)	2 (13%)

## Data Availability

Not applicable.

## Supplementary Materials

The following supporting information can be downloaded at: https://www.mdpi.com/article/10.3390/bios13040419/s1, Video S1: Loading on the chip of a droplet containing a bovine zygote at 48 hpi., Video S2: Movement of a droplet containing an embryo at day 5 of culture., Video S3: Procedure of merging of a droplet containing an embryo with additional droplet of culture medium on day 6 of culture., Table S1: Evaluation of the toxicity of the supplementation of Tween 80 to the culture medium on the in vitro development of bovine embryos., Table S2: Evaluation of the toxicity of the supplementation of Pluronic F127 to the culture medium on the in vitro development of bovine embryos., Figure S1: Schematic representation of the observed effect of the supplementation of bovine embryo culture medium (SOFaa-ITS-BSA) with Pluronic F127 (0.08%) on the droplet shape during group IVC.
