# Supplementary material for: Development of a Microfluidic Chip Powered by EWOD for In Vitro Manipulation of Bovine Embryos
Source: Biosensors (Basel). 2023 Mar 25;13(4):419. doi: 10.3390/bios13040419 (PMC10136516; doi:10.3390/bios13040419)
Supplement: Supplementary file 1 [file biosensors-13-00419-s001.zip › Supplementary material_Development of a microfluidic chip powered by EWOD for in vitro manipulation of bovine embryos.pdf]

biosensors

MDPI

Supplementary material: Development of a microfluidic chip powered by EWOD for in vitro manipulation of bovine embryos

Adriana Karcz [1,2]*, Ann Van Soom [2], Katrien Smits [2], Sandra Van Vlierberghe [3], Rik Verplancke [1], Osvaldo Bogado Pascottini [2], Etienne Van den Abbeel [4] and Jan Vanfleteren [1]*

1 Centre for Microsystems Technology (CMST), Imec and Ghent University, Technologiepark Zwijnaarde 126, 9052 Zwijnaarde, Belgium
2 Reproductive Biology Unit (RBU), Department of Internal Medicine, Reproduction and Population Medicine, Faculty of Veterinary Medicine, Ghent University, Salisburylaan 133 D4, 9820 Merelbeke, Belgium
3 Polymer Chemistry and Biomaterials Group, Centre of Macromolecular Chemistry, Ghent University, Campus Sterre, building S4, Krijgslaan 281, 9000 Ghent, Belgium
4 Department of Human Structure and Repair, Ghent University, Corneel Heymanslaan 10, 9000 Ghent, Belgium
* Correspondence: adriana.karcz@ugent.be (A.K.); jan.vanfleteren@ugent.be (J.V.)

Supplementary material videos

Loading on the chip of a droplet containing a bovine zygote at 48 hpi (S1)

Video S1 shows the procedure of embryo-droplets loading on the chips at 48 hpi. Briefly, a 5 µL droplet containing a zygote is pipetted between the top and bottom plates of the device. Next, the droplet is moved towards the first pair of electrodes by the application of 70 V between the bottom driving electrode and the top electrode connected to the ground.

Movement of a droplet containing an embryo at day 5 of culture (S2)

Video S2 shows the movement of a droplet containing a bovine embryo (presumed non-viable at the time of video acquisition) on day 5 of *in vitro* culture by the application of 70 V between the driving electrode and the top electrode connected to the ground. Black arrow points to the location of the embryo before and after droplet transport.

Procedure of merging of a droplet containing an embryo with additional droplet of culture medium on day 6 of culture (S3)

Video S3 shows the procedure of merging of two droplets on the chip. Briefly, a 7 µL droplet (which was supplied with a 2 µL droplet at 96 hpi in a corresponding manner) containing an embryo was merged with a 2 µL droplet of SOF-ITS-BSA-Tween 80 culture medium on day 6 of culture. The 2 µL droplet was pipetted between the plates in a similar manner as shown is video S1. The 7 µL droplet was moved towards the 2 µL droplet by the application of 94 V to the bottom electrode, the top electrode was connected to the ground. Black arrow points to the position of the embryo, which was presumed non-viable at the time of video acquisition. The speed of the video was increased (2x).

Toxicity tests of Tween 80 and Pluronic F127

The addition of surfactants may affect the interfacial tension between the aqueous droplet and the surrounding oil (Figure S1). In order to evaluate the possible toxic effects of the addition of Tween 80 to the bovine embryo culture medium, a 96-well plate with U-shaped bottom wells was used. In this way, the effect of changed droplet shape in the standard droplet culture in 4-well dishes was avoided. The effect of the culture in U-shaped bottom well on the development of bovine embryos *in vitro* was included in this test. Briefly, bovine embryos were *in vitro* matured and fertilized as described in the Materials and Methods section of the main article. After fertilization, embryos were cultured

in groups of 22-28 in 50 µL of SOFaa-ITS-BSA culture medium (groups 1 and 2) or SOFaa-ITS-BSA culture medium enriched with Tween 80 (0.01 %, group 3). The medium was covered with 150 µL of mineral oil. This test was done in 1 replicate. The cleavage (48 hpi) and blastocyst rates (at 168 and 192 hpi) are presented in Table S1. The results were found to be comparable between the groups and based on these, as well as the known effects of interaction between Tween 80 with BSA and hydrophobic surfaces as described in the Discussion section of the main article, Tween 80 was used in the design and execution of the final study.

Table S1. Evaluation of the toxicity of the supplementation of Tween 80 to the culture medium on the *in vitro* development of bovine embryos.

Group	Group number	Total number of embryos	Cleavage rate [%]	Blastocyst rate 168 hpi (day 7) [%]	Blastocyst rate 192 hpi (day 8) [%]
Control 4-well dish	1	45	78	11	27
U-shaped bottom well control	2	44	89	14	23
U-shaped bottom well with Tween 80 (0.01%)	3	49	84	8	27

Initially, the Pluronic F127 surfactant was considered as a method to protect the surface of the chip from fouling with proteins, namely BSA. This surfactant is considered non-toxic and biocompatible, and was used in [1]. For the evaluation of potential surfactant's toxicity to early embryo development, a test using standard 4-well culture dishes was performed. The embryos were cultured in groups of 22-28 in 50 µL droplets of SOFaa-ITS-BSA culture medium (group 1) and SOFaa-ITS-BSA culture medium enriched with Pluronic F127 (0.08 %, group 2). Upon droplet creation an observation was made, namely that the addition of surfactant affects the shape of droplets of the medium (Figure S1). The blastocyst rates at 192 hpi of the embryos cultured in group 2 were only half of those in the control group. However, this result was found to be inconclusive as it was impossible to directly associate this inferior result with the possible toxic effect of the surfactant, nor the altered droplet shape. This test was performed in 1 replicate. The examination of Pluronic F127 as the candidate in the construction of the microdevice was not followed further.

Table S2. Evaluation of the toxicity of the supplementation of Pluronic F127 to the culture medium on the *in vitro* development of bovine embryos.

Group	Group number	Total number of embryos	Cleavage rate [%]	Blastocyst rate 168 hpi (day 7) [%]	Blastocyst rate 192 hpi (day 8) [%]
Control 4-well dish	1	91	79	14	29
4-well dish with Pluronic F127 (0.08%)	2	104	80	8	14

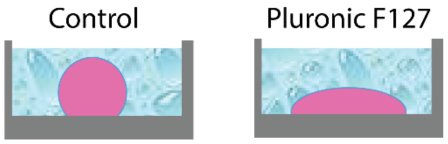

Figure S1. Schematic representation of the observed effect of the supplementation of bovine embryo culture medium (SOFaa-ITS-BSA) with Pluronic F127 (0.08 %) on the droplet shape during group IVC.

[1] H. Y. Huang *et al.*, "Digital Microfluidic Dynamic Culture of Mammalian Embryos on an Electrowetting on Dielectric (EWOD) Chip," *PLoS One*, vol. 10, no. 5, 2015, doi: 10.1371/journal.pone.0124196.
